# Itchy and Swollen: Atopic Dermatitis With Cephalocervical Lymphadenitis in an Infant

**DOI:** 10.7759/cureus.68723

**Published:** 2024-09-05

**Authors:** Nehaa Sohail, Ayaan Sohail, Wasiq Nadeem, Huma Y Lodhi

**Affiliations:** 1 Medical Education, Paul L. Foster School of Medicine, Texas Tech University Health Sciences Center, El Paso, USA; 2 Medical Education, McGovern Medical School, The University of Texas Health Science Center, Houston, USA; 3 Internal Medicine, Keck Medicine, University of Southern California, Los Angeles, USA; 4 Pediatrics, El Paso Kids Klinic, El Paso, USA

**Keywords:** atopic dermatitis in children, cephalocervical lymphadenitis, dermatology, inflammatory skin disorder, pediatrics

## Abstract

We present a case of a three-month-old Hispanic female seen in the clinic for atopic dermatitis (AD) along with a rare incidence of associated cephalocervical lymphadenitis. The patient had a three-month history of redness, irritation, inflammation, and pruritus of the scalp, face, torso, and lymph nodes. The history and examination originally indicated seborrheic dermatitis with AD, impetigo, and folliculitis on the differential. Due to the rarity of this presentation, it is crucial to increase clinical recognition and awareness of this combination among physicians to improve patient outcomes. Recognizing this unusual presentation can lead to more accurate diagnoses and tailored treatment plans, ultimately benefiting the patient while also advancing our understanding of similar cases.

## Introduction

Atopic dermatitis (AD) or eczema is a chronic inflammatory skin disorder that causes redness, irritation, inflammation, and pruritus of the skin in infancy [1]. AD is characterized by redness, swelling, cracking, crusting, clear fluid, and scaling that can appear anywhere on the body. The cause of AD is unknown, but it is believed to be a combination of genetic, environmental, and immune system factors [1]. On the contrary, lymphadenitis denotes inflammation of lymph nodes in a particular region of the body, typically characterized by erythema and warmth. It is often caused by infections, malignancies, or autoimmune disorders and can affect individuals of any age. We describe an exceptionally rare manifestation of AD with associated cephalocervical lymphadenitis.

## Case presentation

**Figure 1:**
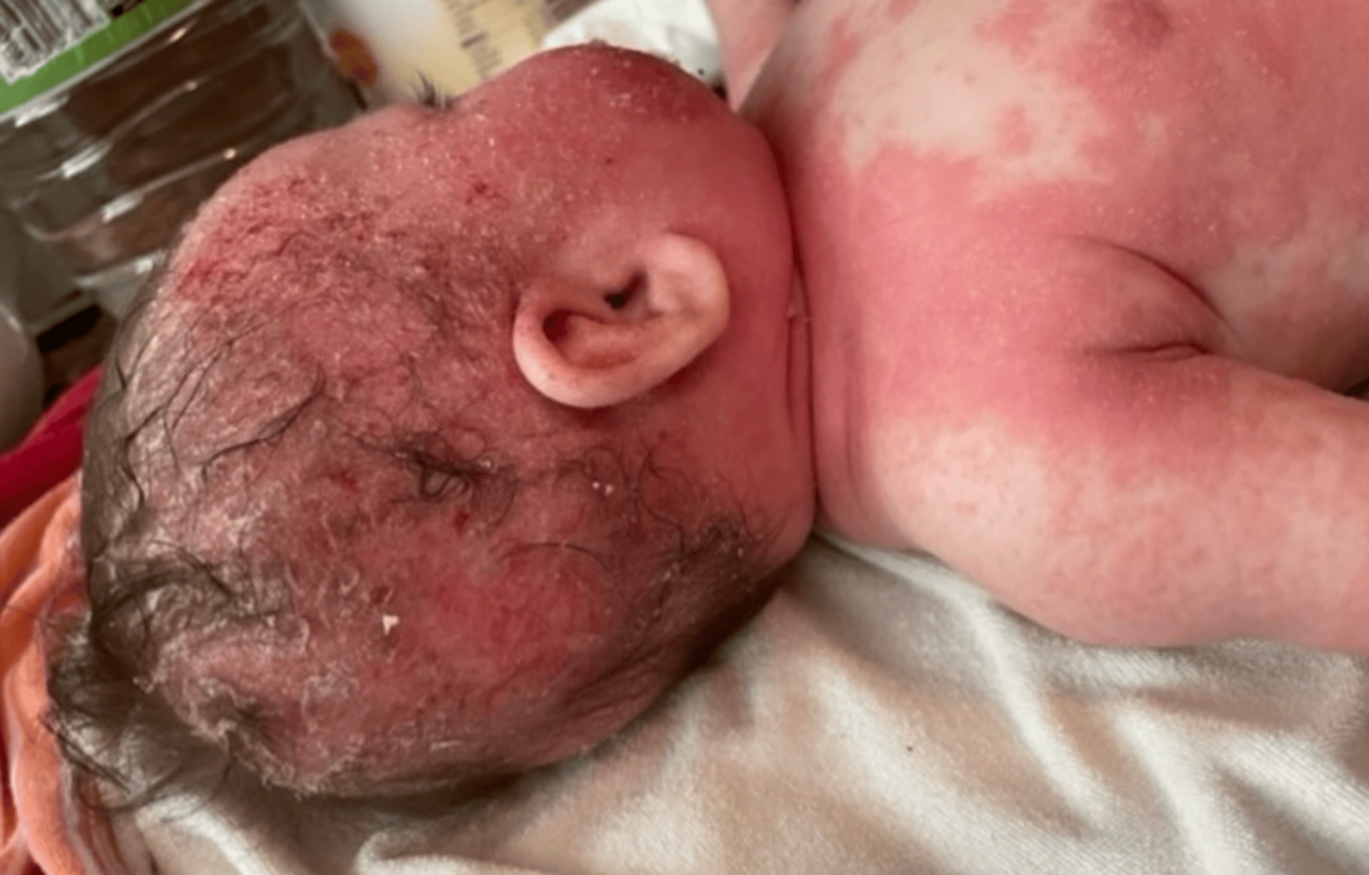
Cephalofacial erythema with dry, crusty scaling and excoriations on the forehead and cheek

**Figure 2:**
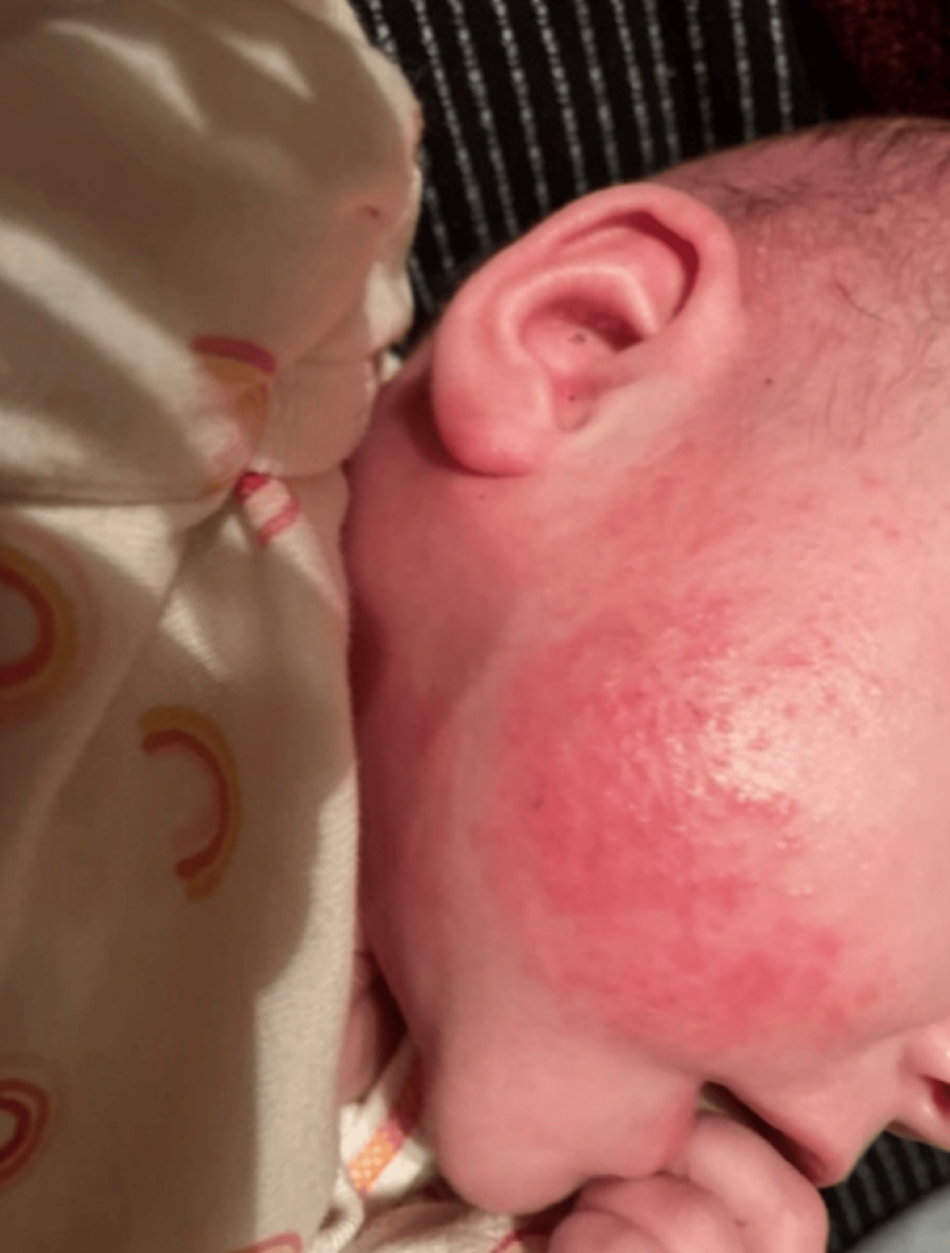
Maculopapular erythema on the cheek with swollen cervical lymph nodes

**Figure 3:**
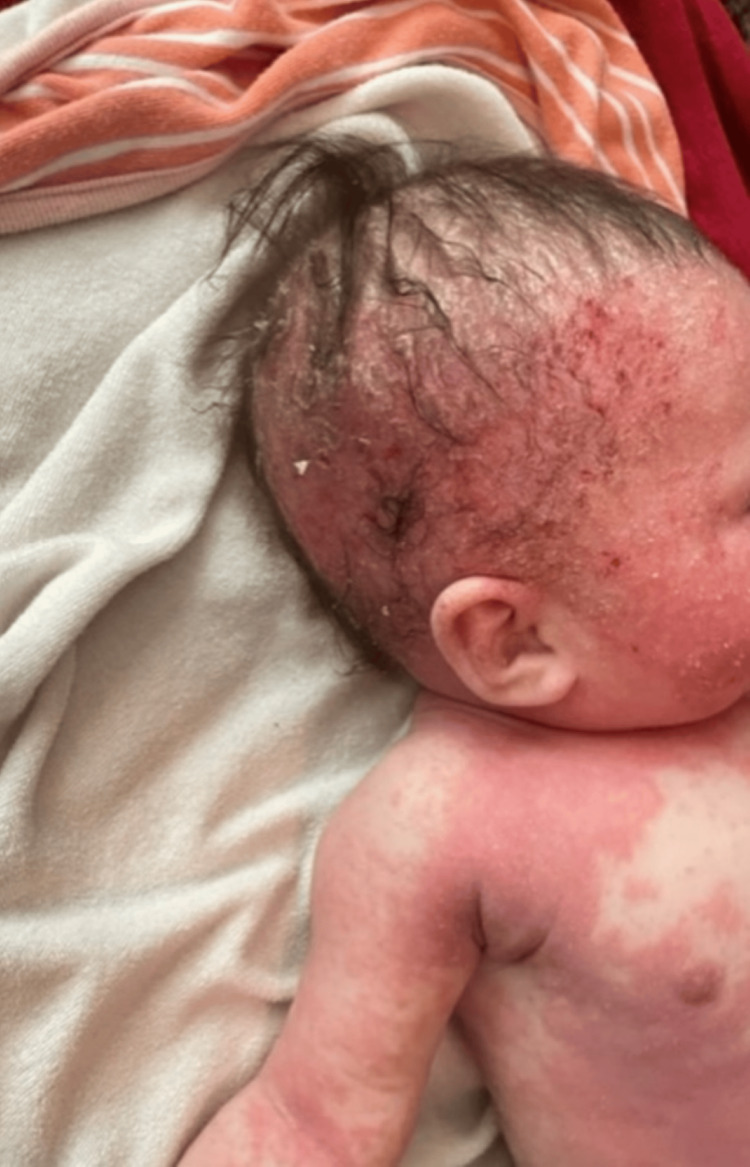
Atopic dermatitis of the scalp, face, and torso with swollen cephalic, auricle, and cervical lymph nodes

A three-month-old Hispanic female originally diagnosed with seborrheic dermatitis presents with AD and associated cephalocervical lymphadenitis. The patient was first seen when she developed a rash on her cheeks. A couple of days later, the rash worsened by spreading all over her body. General symptoms, including fatigue, fussiness, runny nose, congestion, and cough, were also noted. Vital signs were normal. Diagnosis of seborrheic dermatitis was made with treatment options of scalp oils, moisturization with Cetaphil or Aveeno, Happy Cappy Shampoo, and avoidance of triggers. The patient returned to the clinic six days later with redness and pruritus of the skin, along with flaking of the scalp. This pattern was consistent for three weeks with no improvement. General symptoms of congestion and dry cough were also noted. Vital signs stayed normal. The treatment plan was reassurance and continuity of original treatment options. Three months later, the six-month-old patient presented with lymphadenitis of the cephalic, cervical, and auricle lymph nodes. General symptoms of fussiness and sleep disturbance secondary to pruritus were also noted. Physical examination revealed dry, scaly, and crusty skin, mostly on the scalp and face (Figure 1). Swollen lymph nodes were palpable on the scalp, pre- and postauricle, and anterior cervical areas (Figure 2). The patient was diagnosed with moderate to severe AD with 10% total body surface area involvement with swollen lymph nodes (Figure 3).

Initially, the patient was given topical steroids, but the rash worsened. The patient was then referred to a pediatric dermatologist. The history and examination indicated AD, along with a rare incidence of associated cephalocervical lymphadenitis with seborrheic dermatitis, impetigo, and folliculitis on the differential. She was prescribed triamcinolone for the body twice a day for two weeks, hydrocortisone 2.5% for the face and scalp twice a day for a month, and ketoconazole 2% shampoo followed by DermaSmoothe oil for the scalp two to three times a week. Discontinuation of ketoconazole 2% shampoo occurred due to significant irritation. Weekly follow-ups were also recommended. The patient’s condition is currently under control, but flares and weens occur with environmental triggers. This case is a great reminder that not all skin disorders are the same and that AD can occur with other conditions, such as lymphadenitis.

## Discussion

Clinically, AD is a very common inflammatory skin condition in infants and children, affecting 20% of children worldwide. AD is characterized by erythematous papules, vesicles, and weeping lesions, often with excoriations and crusting in the acute phase. Lichenification, dry and scaly plaques, and accentuation of skin marking can occur due to repeated scratching in the chronic phase. AD leads to abnormalities in the epidermis and the immune system and is often associated with other IgE-associated disorders like asthma, food allergens, and allergic rhinitis. The atopic triad (AD, allergic rhinoconjunctivitis, and asthma) may start simultaneously or consecutively in what is known as the “atopic march,” which is the natural progression of allergic diseases in children [1]. The symptomatology is due to a defective barrier of the skin and the respiratory tract [2]. Food hypersensitivity may also cause or exacerbate AD in 10-30% of patients [3]. In all, 90% of such flares or reactions are caused by allergens such as milk, eggs, soy, peanuts, and wheat [4]. AD symptoms may also worsen with exposure to triggers like fabrics, stress, and detergents. The treatment for AD includes managing triggers, maintaining a good skincare routine, anti-inflammatory therapy, applying cream moisturizers, topical corticosteroids, antihistamines, and, in severe cases, using systemic medications or phototherapy [5]. Topical steroids are first-line agents for acute flares and should be given for tapering every other day [2]. Maintaining a good skincare routine includes the application of emollients twice daily within a few minutes of exiting the bath or shower to lock in moisture. Dupilumab is an FDA-approved biologic therapy that blocks the IL-4 receptor and has shown promising results for moderate to severe AD [6]. Some alternative therapies for AD include low-allergen maternal diets and weekly bleach baths to limit *Staphylococcus aureus* colonization, which has shown a 50% decreased frequency of AD from ages one to four years old [7]. Some differential diagnoses include contact dermatitis, psoriasis, seborrheic dermatitis, and other eczematous disorders. Overall, most patients with AD improve over time, with the exception of some cases.

Lymphadenitis, in contrast, refers to inflammation of lymph nodes, usually by infection, malignancy, or autoimmune processes affecting individuals of all ages. Lymphadenitis presents with tender, enlarged lymph nodes in a specific region of the body, often with erythema and warmth. The peak incidence in the pediatric population is unknown. However, many analyses demonstrate that 45-57% of healthy children may have palpable lymph nodes at any time [8]. About 90% of children between the ages of four and eight suffer from lymphadenopathy, which is most commonly caused by infections [9]. The clinical history should consider the patient’s age, associated symptoms, time course of the disease, zoonosis, and any previous travels [10]. When examining the lymph nodes, size and location are important for an accurate diagnosis. Radiologic and laboratory examinations, along with fine-needle aspiration or excisional biopsy, may be used to investigate lymphadenitis. Treatment of lymphadenitis includes managing the underlying condition or with analgesics and abscess drainage [11]. Some differential diagnoses include benign reactive lymphadenopathy, lymphoma, cat scratch disease, and tuberculosis. 

AD typically manifests as localized skin inflammation, but in rare cases, it can be complicated by lymphadenitis, as seen with our patient. Having both conditions occur together is extremely uncommon and should be a point of focus for practicing physicians. Severe eczema can significantly impact the daily lives of patients, leading to pruritus, excessive dryness, discomfort, and inflamed skin. One notable case includes weeping eczema, characterized by fluid-filled blisters that ooze and create a yellow-to-orange crusty layer on the skin after drying [12]. Weeping eczema typically occurs in areas where the skin flexes, such as behind the knees, inside the elbows, and in front of the neck, but it can develop anywhere on the body [12]. Patients with weeping eczema may notice worsening symptoms like swelling and dry, itchy skin and, in severe cases, may experience fatigue, fever, chills, and swollen lymph nodes [12].

Lymphadenitis in the context of eczema often arises due to a secondary bacterial infection, such as impetiginized eczema, where bacteria like *S. aureus* or *Streptococcus pyogenes* infect the eczematous skin, leading to tender and swollen lymph nodes [13]. Viral infections can also cause lymphadenitis, particularly in cases like eczema herpeticum, a severe viral infection caused by the herpes simplex virus, where the immune response to the widespread infection results in inflamed lymph nodes [14]. Though rare, conditions like lymphomatoid papulosis, which mimics lymphoma, can occur in individuals with eczema, resulting in inflammatory nodules on the skin and lymphadenitis [15]. Although these two conditions usually occur separately, it is important to recognize that they may co-occur in some rare cases, as observed in our patient.

## Conclusions

This case highlights the importance of clinical recognition and diagnosis of AD with lymphadenitis among healthcare providers to expedite treatment and mitigate challenges faced by patients. While AD itself may seem straightforward, it can manifest alongside other underlying conditions, including cephalocervical lymphadenitis. However, some factors contributing to misdiagnoses may include patient presentation, age, and delayed or absent consultations. Later diagnoses often correlate with more advanced disease stages and heightened daily burdens and costs for patients. Given the rarity of this combination, augmenting clinical awareness among physicians is essential for enhancing patient outcomes. Identifying this uncommon presentation can improve diagnostic precision and customize treatment strategies, ultimately benefiting the patient and advancing our knowledge of similar cases.
